# Protocol for ex vivo physicochemical assessment of photothermally preconditioned platelet-rich plasma

**DOI:** 10.1016/j.mex.2026.103999

**Published:** 2026-06-07

**Authors:** Laura Cordero, Elena Sánchez-Vizcaíno Mengual, Hernán Pinto

**Affiliations:** Meta Cell Technology, Sant Cugat, Spain

**Keywords:** Red light, Turbidity, pH, Temperature, Regenerative medicine, Sample handling, Paired-sample workflow, Ex vivo assessment

## Abstract

Photothermal biomodulation is used to precondition platelet-rich plasma, but standardized laboratory workflows for monitoring associated physicochemical changes remain limited. This article describes a paired ex vivo protocol for assessing selected physicochemical parameters of platelet-rich plasma before and after photothermal preconditioning.

• Platelet-rich plasma is prepared from donor blood, pooled per donor, and divided into paired control and photothermally preconditioned aliquots.
• The protocol enables time-course monitoring of density/turbidity, pH, and temperature from baseline to 60 min.
• Application in samples from three healthy donors showed that the workflow can detect small time-dependent differences between paired aliquots.

## Graphical abstract

**Figure:**
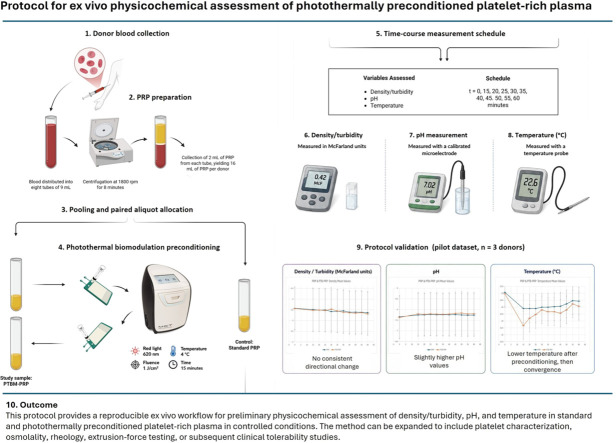

In the pilot validation dataset, photothermally preconditioned samples showed slightly higher pH and lower temperature than paired control samples, whereas density/turbidity showed no consistent directional change. This protocol provides a reproducible ex vivo workflow for preliminary physicochemical assessment of photothermally preconditioned platelet-rich plasma and may be expanded to include platelet characterization, osmolality, rheology, extrusion-force testing, or subsequent clinical tolerability studies.

## Graphical abstract

Paired ex vivo workflow for physicochemical assessment of standard PRP and photothermally preconditioned PRP. Donor-derived PRP is pooled, divided into paired aliquots, and either maintained as standard PRP or exposed to PTBM conditions. Density/turbidity, pH, and temperature are monitored from baseline to 60 min.

**Specifications table**

**Table:** 

**Subject area**	Biochemistry, Genetics and Molecular Biology
**More specific subject area**	Platelet-rich plasma; regenerative medicine; photobiomodulation; ex vivo physicochemical characterization
**Name of your protocol**	Ex vivo physicochemical assessment of photothermally preconditioned platelet-rich plasma
**Reagents/tools**	Peripheral whole blood; 3.8% sodium citrate Vacutainer tubes; platelet-rich plasma; sterile single-use PTBM kits; red-light photothermal biomodulation device; 16-mm glass tubes; McFarland densitometer; portable pH meter with microelectrode; temperature probe; pH 7.0 and pH 10.0 calibration buffers; distilled water; protein-cleaning solution
**Experimental design**	Paired ex vivo protocol using PRP prepared from adult healthy donor blood meeting eligibility criteria. For each donor, PRP is pooled and divided into two paired aliquots: non-preconditioned standard PRP and photothermally preconditioned PRP. The treated aliquot is exposed to red light at 620 nm and 1 J/cm² while maintained at 4 °C for 15 min. Density/turbidity, pH, and temperature are measured at baseline, immediately after preconditioning, and every 5 min until 60 min.
**Trial registration**	Not applicable
**Ethics**	Written informed consent was obtained from all healthy donors for blood donation and research use of derived samples. Samples were anonymized/coded before laboratory analysis. No therapeutic intervention, diagnostic procedure, or experimental treatment was administered. No prior institutional ethics committee approval was obtained; this is acknowledged as a limitation.
**Value of the Protocol**	• This protocol provides a standardized paired-sample workflow for assessing selected physicochemical parameters of platelet-rich plasma before and after photothermal preconditioning. • The within-donor paired design enables direct comparison between standard PRP and photothermally preconditioned PRP while reducing the influence of inter-donor variability. • The workflow enables time-course monitoring of density/turbidity, pH, and temperature under controlled ex vivo conditions. It can be expanded to include platelet characterization, osmolality, rheology, extrusion force testing, growth factor analysis, or clinical tolerability outcomes.

## Background

Platelet-rich plasma (PRP) is an autologous blood-derived product used in several regenerative medicine settings, including dermatology, aesthetic medicine, orthopaedics, ophthalmology, gynaecology, sports medicine, and surgery [1]. Donor-related factors, preparation methods, handling conditions, and preconditioning procedures can influence its composition and behaviour. For this reason, reproducible workflows are needed to characterise PRP under defined ex vivo conditions before its use in more advanced biological, mechanical, or clinical studies.

Photobiomodulation and related light-based preconditioning approaches have been investigated as strategies to modulate cellular and platelet-associated biological activity [[2], [3], [4]]. Photothermal biomodulation (PTBM) combines controlled light exposure with temperature regulation and has been applied to PRP in regenerative and aesthetic medicine contexts [5]. However, preconditioning procedures may also modify basic physicochemical characteristics of PRP, including temperature, pH, turbidity, osmolality, viscosity, or flow behaviour. These variables may be relevant for sample handling, formulation characterisation, injectability assessment, and the design of subsequent translational studies [[6], [7], [8]].

A standardised ex vivo protocol can help researchers assess whether PTBM preconditioning produces measurable changes in PRP under controlled laboratory conditions. A paired-sample design, in which PRP from the same donor is divided into untreated and treated aliquots, is useful because it reduces the influence of inter-donor variability and allows direct comparison between standard PRP and PTBM-PRP.

The protocol described here provides a workflow for preparing PRP from citrate-anticoagulated whole blood, dividing the sample into paired aliquots, applying PTBM preconditioning, and monitoring selected physicochemical parameters over time. The current version focuses on density/turbidity, pH, and temperature measured from baseline to 60 min. The protocol is intended for preliminary ex vivo product characterisation. It can be adapted to include additional endpoints, such as platelet concentration, leukocyte content, erythrocyte contamination, osmolality, rheology, extrusion force testing, growth factor release, or molecular profiling.

The protocol may be useful for laboratories developing or evaluating PRP preconditioning procedures, particularly when a simple, reproducible, paired-sample workflow is required before undertaking more complex mechanistic, injectability, or clinical studies.

## Description of protocol

This protocol describes a paired ex vivo workflow for assessing selected physicochemical parameters of platelet-rich plasma (PRP) before and after photothermal biomodulation (PTBM) preconditioning. The procedure is based on preparing PRP from citrate-anticoagulated whole blood, division of each donor-derived PRP sample into paired control and treated aliquots, application of PTBM to one aliquot, and repeated measurement of density/turbidity, pH, and temperature over a 60-minute observation period.

The paired design allows each donor sample to serve as its own control. This approach reduces the influence of inter-donor variability and enables direct comparison between standard PRP and PTBM-preconditioned PRP under controlled ex vivo conditions.

The protocol includes the following stages:

1. Donor blood collection.
2. PRP preparation.
3. Pooling and paired aliquot allocation.
4. PTBM preconditioning.
5. Time-course measurement schedule.
6. Density/turbidity measurement.
7. pH measurement.
8. Temperature measurement.
9. Time-course data collection.
10. Quality control considerations.
11. Data recording and analysis.

**1. Donor blood collection**

Peripheral whole blood is collected from healthy adult donors after written informed consent has been obtained for blood donation and research use of derived samples.

PRP samples were prepared from peripheral whole blood obtained from healthy adult donors. Inclusion criteria were age ≥18 years, provision of written informed consent for blood donation and research use of derived samples, absence of known acute illness or active infection at the time of sample collection, and availability of sufficient sample volume for the planned ex vivo analyses. Exclusion criteria were known acute infection or clinically relevant inflammatory condition at the time of collection, insufficient sample volume, visible haemolysis, clotting, or suspected sample contamination.

For each donor, collect a total of 64 mL of peripheral whole blood and distribute it into 8 Vacutainer tubes containing 9 mL of 3.8% sodium citrate. In the validation dataset for this protocol, tubes from Sanisus Medical (Castellón, Spain) were used.

After collection, gently invert the tubes according to the manufacturer’s instructions to ensure adequate mixing with the anticoagulant. Avoid vigorous shaking, which may cause haemolysis or unintended platelet activation.

**2. PRP preparation**

Centrifuge the citrate-anticoagulated whole blood tubes at:

1800 rpm for 8 min at room temperature.

After centrifugation, carefully collect the platelet-rich plasma fraction from each tube. In the validation dataset, approximately 2 mL of PRP was collected per tube, yielding approximately 16 mL of PRP per donor.

Transfer the PRP fractions from the same donor into a sterile 20 mL tube without additives and gently mix to obtain a homogeneous donor-specific pooled PRP sample.

**Practical note:** PRP should be handled gently throughout the procedure. Avoid repeated pipetting, strong agitation, or unnecessary delays between centrifugation, aliquoting, and preconditioning.

**3. Pooling and paired aliquot allocation.**

After pooling, divide the PRP obtained from each donor into two equal aliquots of approximately 8 mL each:

• **Control aliquot:** standard non-preconditioned PRP.
• **Treated aliquot:** PRP assigned to PTBM preconditioning.

Transfer each aliquot into a separate 15 mL tube.

This paired allocation should be performed independently for each donor. The control and treated aliquots must be processed in parallel and measured using the same timing schedule.

**Practical note:** The paired aliquot design is central to the protocol. Each treated sample should always be compared with the matched control sample obtained from the same donor.

**4. Photothermal biomodulation preconditioning.**

Transfer approximately 8 mL of the treated PRP aliquot into the sterile single-use PTBM kit compatible with the photothermal biomodulation device.

In the validation dataset, PTBM was performed using the MCT System, including the MCT Unit and sterile single-use MCT Kits, manufactured by Meta Cell Technology, Sant Cugat, Spain.

Apply the PTBM-PRP preset using the following parameters:

**Table:** 

**Parameter**	**Setting**
Light wavelength	620 nm
Fluence	1 J/cm²
Temperature	4 °C
Exposure time	15 min
Sample type	Platelet-rich plasma
Sample volume	Approximately 8 mL

The PTBM parameters used in this protocol correspond to the predefined PRP preconditioning preset of the MCT System. This preset is intended for the photothermal preconditioning of PRP before clinical use and combines low-temperature conditioning with red-light exposure. The present protocol was designed to characterize selected physicochemical properties of PRP after application of this predefined preconditioning program, rather than to compare or optimize different wavelengths, fluences, temperatures, or exposure times.

The control aliquot should remain non-preconditioned and should be maintained under the same general laboratory conditions, except for PTBM exposure.

**Practical note:** The PTBM conditions should be fully reported in any implementation of this protocol. If another device or light source is used, the wavelength, fluence, exposure time, sample volume, cooling conditions, and distance or geometry of light exposure should be documented.

**5. Time-course measurement schedule**

Measure density/turbidity, pH, and temperature in both standard PRP and PTBM-preconditioned PRP at the following time points:

**Table:** 

**Time point**	**Description**
t = 0 min	Baseline, before PTBM preconditioning
t = 15 min	Immediately after completion of PTBM preconditioning
t = 20 min	5 min after PTBM completion
t = 25 min	10 min after PTBM completion
t = 30 min	15 min after PTBM completion
t = 35 min	20 min after PTBM completion
t = 40 min	25 min after PTBM completion
t = 45 min	30 min after PTBM completion
t = 50 min	35 min after PTBM completion
t = 55 min	40 min after PTBM completion
t = 60 min	45 min after PTBM completion

The same time points should be used for both paired aliquots.

**Practical note:** Timing should be standardised across donors. The t = 15 min time point corresponds to the end of the PTBM preconditioning period.

**6. Density/turbidity measurement.**

Density/turbidity is measured in McFarland units using a McFarland densitometer.

In the validation dataset, measurements were performed using a DEN-1 McFarland densitometer (Biosan, Novosibirsk, Russia).

**6.1 Procedure**

a. Switch on the densitometer approximately 45 min before the first measurement to allow stabilisation.
b. Transfer a minimum of 2 mL of PRP into a standardised 16-mm glass tube.
c. Insert the tube into the densitometer according to the manufacturer’s instructions.
d. Record the density/turbidity value in McFarland units.
e. Repeat the measurement at each predefined time point for both standard PRP and PTBM-preconditioned PRP.

**6.2 Data recording**

Record the following information for each measurement:

• Donor code.
• Sample condition: standard PRP or PTBM-PRP.
• Time point.
• McFarland value.
• Operator initials.
• Any visible sample abnormality, such as clotting, haemolysis, sedimentation, or air bubbles.

**Practical note:** Use the same tube type for all measurements. Bubbles, fingerprints, tube scratches, or incomplete sample volume may affect densitometer readings.

**7. pH measurement.** pH is measured using a calibrated pH meter equipped with a microelectrode and temperature probe.

In the validation dataset, pH was measured with a Jenway pH550 portable pH meter (Thermo Fisher Scientific, Waltham, MA, USA). The pH 7.0 and pH 10.0 calibration buffers were selected according to the manufacturer’s instructions because the expected pH range of citrate-anticoagulated PRP was near-neutral to slightly alkaline.

**7.1 Instrument preparation.**

a. Prepare the pH meter approximately 90 min before measurement.
b. Hydrate the microelectrode according to the manufacturer’s instructions.
c. Perform two-point calibration using:

- pH 7.0 buffer solution.
- pH 10.0 buffer solution.

a. Confirm that calibration is acceptable before sample measurement.
b. Rinse the electrode with distilled water between measurements.
c. Store the electrode in a pH 7.0 buffer when not in use.
d. After completion of measurements, soak the electrode in a protein-cleaning solution for 15–30 min.

**7.2 Sample measurement**

a. Use a minimum of 5 mL of PRP for pH measurement.
b. Insert the microelectrode into the PRP sample, ensuring adequate contact with the liquid phase.
c. Allow the reading to stabilise.
d. Record the pH value at each predefined time point.
e. Rinse the electrode with distilled water before measuring the next sample.

**7.3 Data recording**

Record the following information:

• Donor code.
• Sample condition: standard PRP or PTBM-PRP.
• Time point.
• pH value.
• Calibration buffers used.
• Calibration status.
• Operator initials.
• Any technical issues during measurement.

**Practical note:** Protein-rich biological samples can affect electrode performance. Electrode cleaning and consistent calibration are important for reproducible measurements.

**8. Temperature measurement.**

Sample temperature is recorded at the same predefined time points used for density/turbidity and pH. Temperature was measured simultaneously with pH using a Jenway pH550 portable pH meter (Thermo Fisher Scientific, Waltham, MA, USA).

**8.1 Procedure**

a. Use a calibrated temperature probe or temperature sensor.
b. Measure temperature in both the standard PRP and PTBM-preconditioned PRP aliquots.
c. Record the temperature in degrees Celsius.
d. Perform temperature measurement at baseline, immediately after PTBM completion, and every 5 min until 60 min.

**8.2 Data recording**

Record the following information:

• Donor code.
• Sample condition: standard PRP or PTBM-PRP.
• Time point.
• Temperature in °C.
• Ambient laboratory temperature, if available.
• Operator initials.

**Practical note:** Because PTBM includes cooling at 4 °C, temperature should be measured promptly after completion of the preconditioning period. Delays may reduce the observed temperature difference between paired aliquots.

**9. Quality control considerations**

The protocol is intended for preliminary physicochemical characterisation rather than complete PRP biological characterisation. However, standardised handling is required to improve reproducibility.

The following quality-control steps are recommended:

• Use the same anticoagulant type and blood collection tube format for all donors.
• Apply the same centrifugation parameters to all samples.
• Pool PRP fractions from each donor before aliquot allocation.
• Use paired control and treated aliquots from the same donor.
• Apply the same measurement schedule to both aliquots.
• Calibrate the pH meter before measurement.
• Allow the densitometer to stabilise before use.
• Use the same tube type for density/turbidity measurements.
• Record any deviations from the protocol.

If available, future implementations may include additional PRP quality-control parameters, such as platelet concentration, platelet enrichment, leukocyte count, erythrocyte contamination, sterility, or haemolysis assessment.

**10. Data handling and analysis**

For each donor and each sample condition, record density/turbidity, pH, and temperature at all predefined time points.

Data may be summarised as:

• Individual donor values.
• Mean ± standard deviation.
• Paired differences between standard PRP and PTBM-PRP.
• Time-course plots for each parameter.
• Descriptive comparison of baseline and post-preconditioning values.

Because this protocol is intended for preliminary ex vivo characterisation, statistical testing should be interpreted cautiously, particularly when applied to small validation datasets. If inferential analyses are performed, the statistical approach should be prespecified and clearly described.

For small pilot datasets, descriptive analysis and paired differences may be more appropriate than strong reliance on p-values. Additional studies with larger sample sizes are required if the protocol is used to support confirmatory claims.

## Optional protocol extensions

The current protocol focuses on density/turbidity, pH, and temperature. Depending on the research question, the workflow may be expanded to include:

• Platelet concentration.
• Platelet enrichment factor.
• Leukocyte concentration.
• Erythrocyte contamination.
• Platelet activation markers.
• Growth factor concentration or release kinetics.
• Total protein concentration.
• Albumin/protein fraction analysis.
• Osmolality.
• Viscosity.
• Viscoelasticity.
• Extrusion-force testing.
• Flow-rate assessment.
• Sterility testing.
• Molecular profiling.
• Patient-reported pain or tolerability outcomes in subsequent clinical studies.

These extensions should be planned prospectively and reported separately from the core physicochemical protocol.

## Troubleshooting

**Table:** 

**Issue**	**Possible cause**	**Suggested action**
Variable densitometer readings	Air bubbles, tube scratches, fingerprints, insufficient volume, sedimentation	Use standardised clean tubes, remove bubbles, ensure minimum volume, gently mix sample before measurement
Unstable pH readings	Inadequate electrode hydration, protein contamination, poor calibration, insufficient sample volume	Hydrate the electrode, repeat calibration, clean the electrode with protein-cleaning solution, and confirm adequate sample volume
Rapid temperature convergence between groups	Delay after PTBM completion, high ambient temperature, small sample volume	Measure immediately after PTBM, standardise timing, record ambient conditions
Visible clotting	Sample activation, delayed processing, inadequate anticoagulant mixing	Exclude affected sample, document deviation, standardise blood tube inversion and processing time
Inconsistent paired comparison	Unequal aliquot volumes, incomplete pooling, different handling times	Pool donor PRP before aliquoting, divide into equal volumes, process paired aliquots in parallel

## Expected output

The expected output of the protocol is a time-course dataset containing density/turbidity, pH, and temperature values for paired standard PRP and PTBM-preconditioned PRP aliquots from each donor.

The protocol should generate.

• A paired dataset for each donor.
• Mean and standard deviation values for each parameter.
• Time-course plots for standard PRP and PTBM-PRP.
• Descriptive information on whether PTBM preconditioning is associated with measurable changes in selected physicochemical parameters under the tested conditions.

This output can be used as a preliminary characterisation dataset and may inform the design of future studies incorporating broader PRP characterisation, injectability testing, biological assays, or clinical endpoints.

## Protocol validation

The protocol was applied to platelet-rich plasma samples prepared from three healthy adult donors aged 28–46 years. Three donors were used for pilot protocol validation to assess feasibility, reproducibility of workflow execution, and generation of paired time-course data. This number was not intended to provide statistical validation or to capture the full biological variability of PRP. For each donor, PRP was prepared from citrate-anticoagulated whole blood, pooled, and divided into paired aliquots: one was maintained as standard PRP, and the other underwent photothermal preconditioning with red light at 620 nm, 1 J/cm², and 4 °C for 15 min. Density/turbidity, pH, and temperature were then measured at baseline, immediately after preconditioning, and every 5 min until 60 min. This pilot application was used to verify that the protocol could be performed reproducibly and could generate time-course physicochemical data from paired PRP aliquots.

**a. Density/turbidity.**

**Fig. 1:**
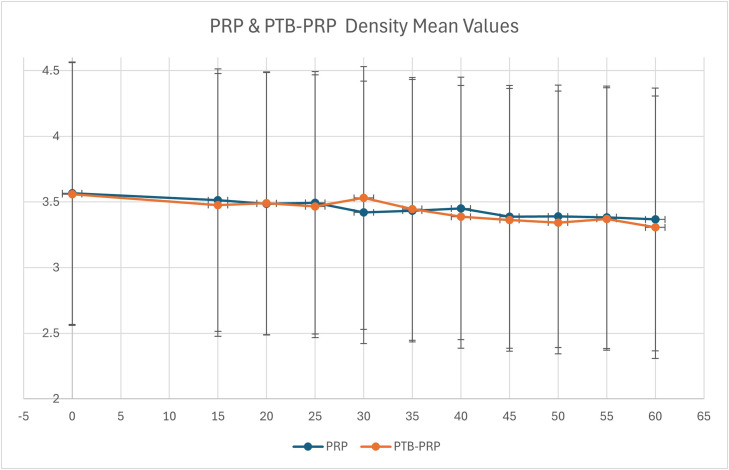
Density/turbidity (McFarland units) of standard PRP and PTBM-PRP over time. Data are presented as mean ± SD (n = 3 donors). Mean density/turbidity values are shown for both groups from baseline (t = 0 min) to t = 60 min.

Density/turbidity was measured in McFarland units from baseline to 60 min. At baseline, standard PRP and the aliquots assigned to PTBM preconditioning showed similar values. Across the observation period, density/turbidity values remained within a narrow range in both conditions. In this pilot application, PTBM-preconditioned PRP did not show a consistent directional change in density/turbidity compared with paired standard PRP (Fig. 1).

These findings indicate that the protocol can be used to monitor time-dependent turbidity/density values in paired PRP samples. However, density/turbidity should not be interpreted as a direct measure of injectability, viscosity, platelet concentration, or platelet activation. Future applications may combine this protocol with rheological measurements, extrusion force testing, and platelet characterisation.

**Fig. 2:**
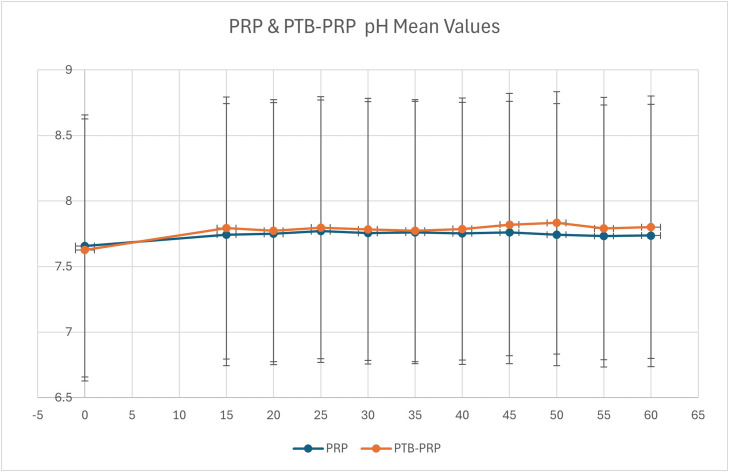
pH values of standard PRP and PTBM-PRP over time. Data are presented as mean ± SD (n = 3 donors). Mean pH values are shown for both groups from baseline (t = 0 min) to t = 60 min.

**b. pH.** pH was measured at the same predefined time points. Baseline pH values were similar between standard PRP and aliquots assigned to PTBM preconditioning. During the post-preconditioning observation period, PTBM-preconditioned PRP showed slightly higher pH values than paired standard PRP. In the pilot dataset, mean pH values remained within a narrow range throughout the 60-minute observation period (Fig. 2).

This confirms that the protocol is suitable for detecting small pH differences between paired PRP aliquots over time. Because the validation dataset included only three donors, the observed pH differences should be considered descriptive and exploratory rather than confirmatory.

**c. Temperature.**

**Fig. 3:**
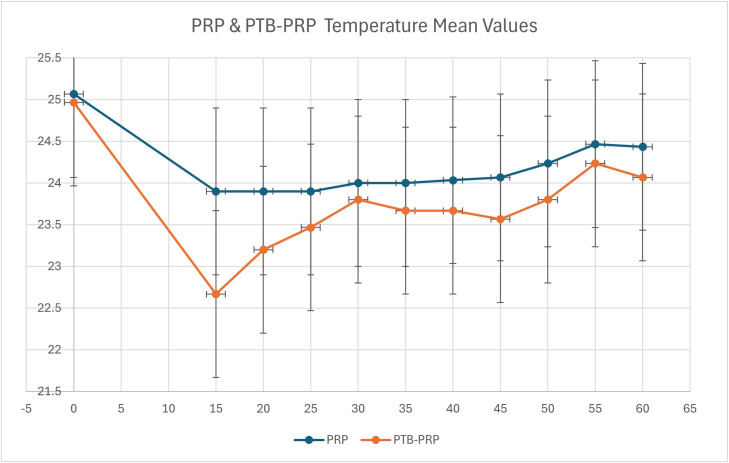
Temperature ( °C) of standard PRP and PTBM-PRP over time. Data are presented as mean ± SD (n = 3 donors). Mean temperature values are shown for both groups from baseline (t = 0 min) to t = 60 min.

Temperature measurements confirmed that the protocol can detect the expected thermal effect of PTBM preconditioning. Immediately after the 15-minute preconditioning period at 4 °C, PTBM-preconditioned PRP showed lower temperature values than paired standard PRP. Temperatures subsequently converged during the observation period, with both conditions showing similar values from later time points onward (Fig. 3).

This pattern supports the feasibility of the time-course design for monitoring temperature changes after PTBM. Because temperature differences may decrease rapidly after preconditioning, prompt measurement after completion of the PTBM cycle is recommended.

## Summary of validation dataset

Application of the protocol to this small pilot dataset demonstrated that the workflow can generate paired time-course measurements of density/turbidity, pH, and temperature in standard PRP and in PRP preconditioned with PTBM. The validation dataset showed:

• Comparable baseline values between paired aliquots before PTBM preconditioning.
• Detectable post-preconditioning differences in pH and temperature.
• No consistent directional change in density/turbidity.
• Feasibility of repeated measurements up to 60 min under controlled ex vivo conditions.

These data support the practical use of the protocol as a preliminary physicochemical characterisation workflow for PTBM-preconditioned PRP. The validation dataset should not be interpreted as evidence of clinical efficacy, improved injectability, or reduced injection-related discomfort. Larger studies incorporating platelet characterisation, osmolality, rheology, extrusion-force testing, and clinical endpoints would be required to evaluate these questions.

Exploratory paired comparisons in this small validation dataset suggested differences in post-preconditioning pH and temperature, but these analyses were not intended to support confirmatory statistical inference.

**Table:** 

**Parameter**	**Baseline comparison**	**Post-preconditioning observation**	**Interpretation for protocol validation**
**Density/turbidity**	Similar between paired aliquots	No consistent directional change after PTBM	Protocol can track turbidity/density over time, but this does not directly measure injectability
**pH**	Similar between paired aliquots	Slightly higher values in the PTBM-preconditioned PRP	The protocol can detect small pH differences between paired aliquots
**Temperature**	Similar between paired aliquots	Lower temperature immediately after PTBM, followed by convergence over time	Protocol can detect the thermal effect of PTBM and subsequent equilibration

To provide additional descriptive information on inter-donor variability, coefficients of variation were calculated for density/turbidity, pH, and temperature at each time point and for each sample condition. Across the validation dataset, CV values were 13.81–15.35% for PTBM-PRP density/turbidity and 12.68–16.18% for standard PRP density/turbidity. For pH, CV values were 0.07–0.83% for PTBM-PRP and 0.93–1.49% for standard PRP. For temperature, CV values were 0.65–5.39% for PTBM-PRP and 0.42–1.26% for standard PRP. A summary of CV values is provided in Supplementary Table 3.

## Limitations

This protocol has several limitations. First, the validation dataset was obtained from a small number of donors and was intended to demonstrate feasibility and data generation rather than to support confirmatory statistical inference. Therefore, the pilot validation data should be interpreted descriptively.

Second, the current protocol evaluates only selected physicochemical parameters: density/turbidity, pH, and temperature. It does not include broader PRP characterisation, such as platelet concentration, platelet enrichment, leukocyte content, erythrocyte contamination, platelet activation markers, growth factor release, sterility testing, or molecular profiling. As a result, the protocol cannot determine whether PTBM preconditioning modifies the cellular or molecular composition of PRP. Furthermore, the protocol does not allow assessment of donor-to-donor variability in platelet yield or the relationship between platelet concentration and the measured physicochemical parameters.

Third, density/turbidity measured in McFarland units should not be interpreted as a direct measure of viscosity, flow behaviour, injectability, or extrusion force. Additional rheological testing and standardised injection-force measurements would be required if the protocol is used to support studies focused on mechanical injectability.

Fourth, the protocol does not include osmolality assessment. Since osmolality may influence formulation behaviour and local tissue response, future adaptations may incorporate osmolality measurement as an additional physicochemical endpoint. Total protein concentration was not measured before or after PTBM preconditioning. Therefore, the protocol cannot determine whether PTBM modifies the protein content of PRP.

Fifth, the protocol is ex vivo and does not reproduce all anatomical, procedural, or patient-related factors that may influence PRP administration in clinical practice. No patient-reported outcomes, pain scores, treatment tolerability assessments, or clinical endpoints are included. Therefore, the validation dataset should not be interpreted as evidence of improved clinical tolerability, reduced injection-related discomfort, or therapeutic efficacy.

Sixth, the PTBM parameters described in this protocol were applied using a specific commercial device and sterile single-use kit. Although the relevant preconditioning parameters are reported, including wavelength, fluence, temperature, exposure time, and sample volume, laboratories using other devices should verify the equivalence of light delivery, thermal control, sample geometry, and exposure conditions before comparing results.

Finally, although written informed consent for blood donation and research use was obtained and samples were anonymised/coded before laboratory analysis, no prior institutional ethics committee approval was obtained for the validation dataset. This should be considered when interpreting and reusing the validation data.

## CRediT author statement

Laura Cordero: Conceptualization, Methodology, Validation, Formal analysis, Investigation, Resources, Data curation, Supervision, Writing – review & editing.

Elena Sánchez-Vizcaíno Mengual: Investigation, Data curation, Writing – original draft, Writing – review & editing.

Hernán Pinto: Conceptualization, Methodology, Validation, Formal analysis, Investigation, Resources, Supervision, Writing – review & editing.

## Declaration of generative AI and AI-assisted technologies in the manuscript preparation process

During the preparation of this work, the author E.S-V.M. used ChatGPT to review the manuscript to detect inconsistencies or duplicates. After using this tool, the author reviewed and edited the content as needed and takes full responsibility for the content of the published article.

## Declaration of competing interest

The authors declare the following financial interests/personal relationships which may be considered as potential competing interests: Laura Cordero and Elena Sánchez-Vizcaíno Mengual are employees of Meta Cell Technology. Hernán Pinto is Chief Scientific Officer of Meta Cell Technology and holds a leadership role in the company. Meta Cell Technology provided the equipment used for the physicochemical analyses and will cover the article processing charge if the manuscript is accepted for publication. The authors received no additional personal fees for this work.

## Data Availability

Data will be made available on request.

## Appendix Supplementary materials

Image, application 1
