# Supplementary material for: Protocol for ex vivo physicochemical assessment of photothermally preconditioned platelet-rich plasma
Source: MethodsX. 2026 Jun 7;17:103999. doi: 10.1016/j.mex.2026.103999 (PMC13273894; doi:10.1016/j.mex.2026.103999)
Supplement: Supplementary file 1 [file mmc1.docx]

# **Supplementary material *and/or* additional information**

**Supplementary Table 1.** Density/turbidity (McFarland units) of standard PRP and PTBM-PRP over time (mean ± SD).

| **Time_min** | **Standard PRP (McF)** | **PTBM-PRP (McF)** |
| --- | --- | --- |
| 0 | 3.57 ± 0.45 | 3.56 ± 0.49 |
| 15 | 3.51 ± 0.49 | 3.48 ± 0.50 |
| 20 | 3.49 ± 0.56 | 3.49 ± 0.49 |
| 25 | 3.49 ± 0.51 | 3.47 ± 0.49 |
| 30 | 3.42 ± 0.55 | 3.53 ± 0.52 |
| 35 | 3.43 ± 0.50 | 3.45 ± 0.49 |
| 40 | 3.45 ± 0.49 | 3.39 ± 0.50 |
| 45 | 3.39 ± 0.53 | 3.36 ± 0.49 |
| 50 | 3.39 ± 0.51 | 3.34 ± 0.50 |
| 55 | 3.38 ± 0.53 | 3.37 ± 0.51 |
| 60 | 3.37 ± 0.47 | 3.31 ± 0.51 |
| Measurements were obtained at baseline (t = 0 min), immediately after PTBM preconditioning (t = 15 min), and every 5 minutes thereafter until t = 60 min.  **Abbreviations:** min, minutes; PRP, platelet-rich plasma; PTBM, photothermal biomodulation; McF: McFarland units. | | |

**Supplementary Table 2.** pH and temperature of standard PRP and PTBM-PRP over time (mean ± SD).

| **Time_min** | **Standard PRP (pH)** | **PTBM-PRP (pH)** | **Standard PRP (°C)** | **PTBM-PRP (°C)** |
| --- | --- | --- | --- | --- |
| 0 | 7.66 ± 0.10 | 7.65 ± 0.05 | 25.07 ± 0.15 | 24.97 ± 0.45 |
| 15 | 7.74 ± 0.08 | 7.79 ± 0.01 | 23.90 ± 0.10 | 22.67 ± 1.22 |
| 20 | 7.75 ± 0.09 | 7.77 ± 0.06 | 23.90 ± 0.10 | 23.20 ± 0.72 |
| 25 | 7.77 ± 0.08 | 7.80 ± 0.01 | 23.90 ± 0.30 | 23.47 ± 0.31 |
| 30 | 7.76 ± 0.07 | 7.78 ± 0.01 | 24.00 ± 0.17 | 23.80 ± 0.17 |
| 35 | 7.76 ± 0.10 | 7.77 ± 0.01 | 24.00 ± 0.17 | 23.67 ± 0.23 |
| 40 | 7.75 ± 0.09 | 7.79 ± 0.01 | 24.03 ± 0.25 | 23.67 ± 0.15 |
| 45 | 7.76 ± 0.10 | 7.82 ± 0.03 | 24.07 ± 0.25 | 23.57 ± 0.15 |
| 50 | 7.74 ± 0.09 | 7.83 ± 0.02 | 24.23 ± 0.23 | 23.80 ± 0.20 |
| 55 | 7.73 ± 0.10 | 7.79 ± 0.05 | 24.47 ± 0.15 | 24.23 ± 0.35 |
| 60 | 7.74 ± 0.12 | 7.80 ± 0.03 | 24.43 ± 0.15 | 24.07 ± 0.31 |
| Measurements were obtained at baseline (t = 0 min), immediately after PTBM preconditioning (t = 15 min), and every 5 minutes thereafter until t = 60 min.  **Abbreviations:** min, minutes; PRP, platelet-rich plasma; PTBM, photothermal biomodulation; ºC, degrees Celsius. | | | | |

**Supplementary Table 3.** Summary of coefficient of variation values for density/turbidity, pH, and temperature across the three donors by parameter and sample condition.

| **Parameter** | **Condition** | **CV minimum (%)** | **CV maximum (%)** | **CV mean (%)** |
| --- | --- | --- | --- | --- |
| Density/turbidity | PTBM-PRP | 13.81 | 15.35 | 14.53 |
| Density/turbidity | Standard PRP | 12.68 | 16.18 | 14.79 |
| pH | PTBM-PRP | 0.07 | 0.83 | 0.31 |
| pH | Standard PRP | 0.93 | 1.49 | 1.18 |
| Temperature | PTBM-PRP | 0.65 | 5.39 | 1.65 |
| Temperature | Standard PRP | 0.42 | 1.26 | 0.77 |
| **Abbreviations:** CV, coefficient of variation; %, percentage; PTBM-PRP, photothermal biomodulated platelet-rich plasma. | | | | |
